# Impact of Coexisting Adenomyosis on the Histopathological, Immunohistochemical, and Molecular Features of Endometrial Cancer and on Adjuvant Therapy Allocation: A Single‐Center Retrospective Cohort Study

**DOI:** 10.1155/ogi/3792144

**Published:** 2026-11-08

**Authors:** Marco D’Asta, Caterina Monaco, Ferdinando Antonio Gulino, Carla Ettore, Giosuè Giordano Incognito, Giuseppe Ettore

**Affiliations:** ^1^ Obstetrics and Gynecology Unit Maternal Child Department ARNAS Garibaldi Nesima Catania Italy; ^2^ Unit of Gynecology and Obstetrics Department of Human Pathology of Adults and Developmental Age “G. Martino” University Hospital Messina Italy

**Keywords:** adenomyosis, adjuvant therapy, endometrial neoplasms, molecular classification, neoplasm staging, retrospective cohort study

## Abstract

**Background and Aims:**

Adenomyosis is frequently found in hysterectomy specimens of women with endometrial cancer (EC), but its relationship with tumor characteristics remains debated. This study aimed to compare the histopathological, immunohistochemical (IHC) and molecular features of EC, and the allocation of adjuvant therapy, between patients with and without coexisting adenomyosis.

**Methods:**

Single‐center retrospective cohort study of consecutive women who underwent hysterectomy for EC at ARNAS Garibaldi Nesima, Catania, Italy, between January 2023 and September 2024, with complete IHC and molecular profiling. Adenomyosis was defined histopathologically. FIGO 2023 stage, histotype and grade, myometrial invasion, lymphovascular space invasion (LVSI), nodal status, p53 and p16 expression, POLE, mismatch repair (MMR), KRAS and PIK3CA status, and adjuvant therapy were compared using Fisher’s exact and chi‐square tests, with odds ratio (OR) and 95% confidence interval (CI).

**Results:**

Of 87 patients, 27 (31.0%) had coexisting adenomyosis. Adenomyosis was associated with FIGO stage I (81.5% vs. 46.7%; OR 5.03, 95% CI 1.68–15.04; *p* = 0.003), myometrial invasion < 50% (66.7% vs. 35.0%; OR 3.71, 95% CI 1.42–9.70; *p* = 0.010), absent/focal LVSI (77.8% vs. 50.0%; OR 3.50, 95% CI 1.24–9.89; *p* = 0.019) and a lower proportion of grade 3 endometrioid tumors (3.7% vs. 26.7%; *p* = 0.017). No differences were observed in nodal status or in p53, p16, POLE, MMR, KRAS, or PIK3CA status. Fewer patients with adenomyosis received adjuvant therapy (29.6% vs. 58.3%; OR 0.30, 95% CI 0.11–0.80; *p* = 0.020). Multivariable adjustment was not feasible given the sample size.

**Conclusion:**

In this cohort, coexisting adenomyosis was associated with earlier‐stage, less invasive EC and, consistently, with less frequent allocation to adjuvant therapy. These are univariate associations from a small, single‐center retrospective series without survival data; they do not establish an independent or causal effect of adenomyosis and require confirmation in larger prospective multicenter studies.

## 1. Introduction

Endometrial cancer (EC) is the most common gynecological malignancy in developed countries, with a rising incidence due to increasing life expectancy and the growing prevalence of risk factors such as obesity and metabolic disorders [1]. Traditionally, EC has been classified into two broad categories based on histopathological and molecular characteristics. Type I tumors, which are primarily endometrioid, are estrogen‐dependent and generally associated with a favorable prognosis. In contrast, type II tumors, which include serous and clear cell carcinomas, are typically estrogen‐independent and display more aggressive behavior [2]. Advances in molecular classification, particularly with The Cancer Genome Atlas (TCGA) and ProMisE systems, have allowed for more precise risk stratification, identifying specific molecular subtypes that influence prognosis and treatment strategies [3, 4].

Adenomyosis is a benign gynecological condition characterized by the presence of ectopic endometrial glands and stroma within the myometrium [5]. It is commonly diagnosed in women of reproductive age and perimenopausal women. Although adenomyosis is frequently identified incidentally in hysterectomy specimens [6], its biological behavior and possible role in tumorigenesis remain unclear. Studies suggest that adenomyosis is found in approximately one in four patients with EC, leading to an ongoing debate about whether it influences tumor progression, prognosis, or response to treatment [7].

The coexistence of EC and adenomyosis has been the subject of numerous investigations, yielding conflicting results regarding their interaction. Several mechanisms have been proposed to explain the potential relationship between these conditions. One hypothesis suggests that both EC and adenomyosis share a common hormonal milieu, as both are estrogen‐dependent disorders. Altered estrogen and progesterone receptor expression in adenomyotic tissue has been reported, potentially influencing the behavior of coexisting EC [8]. Another theory highlights the histopathological and structural interactions between EC and adenomyosis [6, 9]. It has been observed that adenomyosis is involved in 49.5%–53.6% of hysterectomy specimens containing EC, with tumor invasion into adenomyotic foci being attributed to the lower resistance of adenomyotic tissue compared to the endometrial–myometrial junction. This allows adenocarcinoma cells to infiltrate pre‐existing adenomyotic lesions more easily [10]. However, in some cases, true myometrial invasion arises from malignant foci within adenomyosis itself, with irregular clusters of cancer cells extending into the surrounding myometrium, typically accompanied by desmoplastic stroma, edema, and chronic inflammation in the absence of normal endometrial stroma [11–13]. Beyond the structural and hormonal factors, the inflammatory microenvironment of adenomyosis has also been proposed as a key element in its relationship with EC. Chronic inflammation is a characteristic feature of adenomyotic tissue, with increased levels of pro‐inflammatory cytokines and immune‐modulating factors observed in affected areas. Some researchers speculate that this persistent inflammatory response may serve as a barrier to tumor invasion, potentially limiting EC progression. Others argue that chronic inflammation could create a pro‐tumorigenic environment, facilitating malignant transformation [3, 14]. Additionally, emerging evidence suggests that adenomyotic tissues may harbor genetic and epigenetic alterations similar to those observed in EC, including mutations in key oncogenic pathways such as PTEN, KRAS, and PI3K/AKT, further supporting a potential biological link between the two conditions [9]. Despite the frequent coexistence of EC and adenomyosis, their prognostic relationship remains controversial. Some studies proposed that adenomyosis may act as a protective factor, conferring a more favorable prognosis in EC patients [14–18]. However, other studies challenge this notion, proposing that adenomyosis may function as a premalignant lesion, particularly in atypical hyperplasia or where molecular markers suggest a clonal relationship between EC and adenomyotic foci [11, 19]. Endometrial carcinoma arising within adenomyosis (EC‐AIA) is a rare but clinically relevant entity, accounting for approximately 1% of EC cases. The differentiation between EC invading adenomyosis and EC originating within adenomyotic foci is crucial, as it may influence tumor staging and subsequent therapeutic decisions [20–24].

In this context, despite the growing discussion regarding the biological and clinical implications of adenomyosis in EC patients, several clinically relevant issues remain incompletely defined. In particular, the extent to which the coexistence of adenomyosis correlates with contemporary risk stratification, integrating histopathological, immunohistochemical (IHC), and molecular features, remains unclear. Furthermore, limited data are available regarding the potential impact of adenomyosis on treatment‐related outcomes, especially on adjuvant therapy allocation.

Therefore, the aim of the present study was to compare the histopathological, IHC, and molecular characteristics of EC between patients with and without coexisting adenomyosis, and to explore whether the presence of adenomyosis is associated with differences in guideline‐based risk stratification and in the subsequent allocation of adjuvant therapy.

## 2. Materials and Methods

### 2.1. Study Design and Ethical Approval

This was a single‐center retrospective observational cohort study conducted at the Obstetrics and Gynecology Unit, Maternal Child Department, ARNAS Garibaldi Nesima, Catania, Italy. The medical records of all consecutive patients who underwent surgical treatment for EC via hysterectomy between January 2023 and September 2024 were reviewed. The study was conducted in accordance with the ethical principles of the Declaration of Helsinki and is reported following the Strengthening the Reporting of Observational Studies in Epidemiology (STROBE) Statement. Owing to the retrospective design and the use of anonymized data obtained during routine clinical care, the requirement for study‐specific informed consent was waived; all patients had provided written informed consent for surgery and for the use of their anonymized clinical data for research purposes at the time of hospital admission.

### 2.2. Study Population

The study included women aged 18 years or older who had undergone hysterectomy for EC. Only patients with a histologically confirmed diagnosis of EC, with or without concurrent adenomyosis, were considered eligible. The surgical procedures included hysterectomy, with or without bilateral salpingo‐oophorectomy, performed using a minimally invasive approach, and the presence or absence of sentinel lymph node biopsy (SLNB) or pelvic/para‐aortic lymphadenectomy was documented. SLNB was performed in patients with presumed uterine‐confined EC, based on preoperative biopsy findings (histology and grade), preoperative imaging assessment (myometrial invasion and nodal status), and intraoperative evaluation (absence of suspicious extrauterine or nodal disease). When performed, sentinel lymph nodes underwent ultrastaging according to standard pathological procedures. Performance status, evaluated at the time of surgery according to the Eastern Cooperative Oncology Group (ECOG) classification, was also reviewed, and only patients with a performance status of 0 or 1 were included.

Cases in which neoadjuvant therapy had been administered before surgery were excluded to avoid potential alterations in histopathological and molecular tumor characteristics. Similarly, cases in which the uterus was secondarily involved by metastases from another primary tumor site were not considered. High‐risk uterine malignancies such as uterine sarcomas, including carcinosarcomas, were excluded from the analysis due to their distinct biological behavior and prognostic implications. Incomplete surgical staging or missing data that prevented a proper classification of the cases led to further exclusions. Finally, only cases with a complete IHC evaluation and molecular/genomic profiling were included, to allow an integrated classification of every tumor. The flow of patients through the study, with the number of exclusions and their reasons, is shown in Figure 1.

**FIGURE 1:**
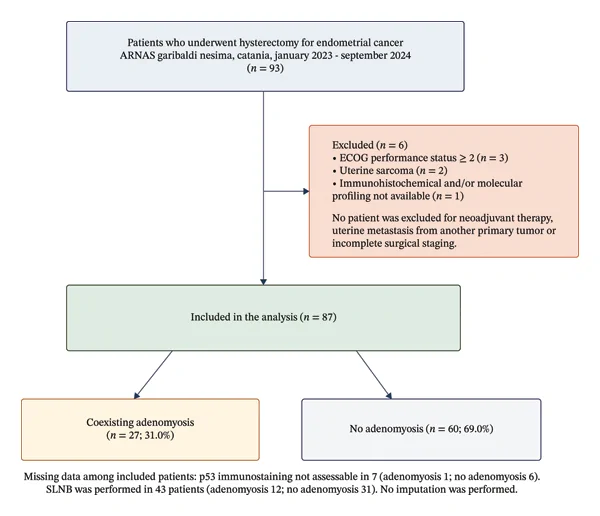
Flow diagram of patient selection. Of 93 patients who underwent hysterectomy for endometrial cancer during the study period, 6 were excluded (ECOG performance status ≥ 2, *n* = 3; uterine sarcoma, *n* = 2; immunohistochemical and/or molecular profiling not available, *n* = 1), leaving 87 patients for analysis. SLNB, sentinel lymph node biopsy.

### 2.3. Histopathological, IHC and Molecular Assessment

All surgical specimens were examined at the institutional Pathology Unit by pathologists with dedicated expertise in gynecological pathology as part of routine diagnostic reporting. Adenomyosis was defined as the presence of ectopic endometrial glands and stroma within the myometrium at the final histopathological examination of the hysterectomy specimen, irrespective of its extent. Patients were categorized into two groups according to the presence or absence of adenomyosis: the first group included patients in whom adenomyosis was identified in association with EC, while the second group comprised patients with EC alone. Diagnoses were made in the context of routine clinical reporting and were not subjected to a study‐specific, blinded second‐reader consensus review; this is acknowledged as a limitation.

Histopathological classification of EC was based on established morphological criteria and an integrated IHC and molecular assessment. Tumor histologic subtypes were primarily assigned based on conventional morphological features, while IHC staining was used systematically to support subtype classification in cases with ambiguous or overlapping morphology. In this context, p53 immunostaining patterns (wild‐type versus aberrant overexpression or null pattern) and p16 expression were used to aid in distinguishing endometrioid from nonendometrioid carcinomas, including serous carcinoma. Mismatch repair (MMR) protein immunostaining and/or microsatellite instability (MSI) assessment were performed to identify MMR‐deficient (MMRd) tumors, and POLE exonuclease‐domain sequencing was used to identify ultramutated (POLEmut) tumors. KRAS and PIK3CA alterations were also recorded. The surrogate molecular class (POLEmut, MMRd, p53‐abnormal [p53abn], or no specific molecular profile [NSMP]) was assigned hierarchically in this order, according to the current recommendations [4, 25].

The clinical and pathological data extracted from medical records included patient age at the time of surgery, stage, tumor histological type and grade, depth of myometrial invasion, presence or absence of lymphovascular space invasion (LVSI, classified as absent/focal or substantial), results of SLNB when performed, findings from pelvic and para‐aortic lymphadenectomy, IHC and molecular markers, and information on adjuvant treatments, including vaginal brachytherapy, external beam radiotherapy, chemotherapy, and combined modalities. Tumor classification followed the criteria defined by the American Joint Committee on Cancer (AJCC) and the Union for International Cancer Control (UICC) TNM classification (8th edition) [26], and surgical–pathological staging was performed according to the 2023 International Federation of Gynecology and Obstetrics (FIGO) staging system [27].

### 2.4. Risk Stratification and Adjuvant Treatment Allocation

Each patient was assigned to one of five prognostic risk groups—low risk, intermediate risk, high‐intermediate risk, high risk, and advanced/metastatic disease—according to the ESGO/ESTRO/ESP 2021 guidelines for the management of patients with endometrial carcinoma [25], which integrate FIGO stage, histotype and grade, depth of myometrial invasion, LVSI status, and the surrogate molecular class (Figure 2). Briefly, low‐risk disease comprised stage I–II POLEmut tumors without residual disease and stage IA MMRd/NSMP low‐grade endometrioid tumors with absent or focal LVSI; intermediate risk comprised stage IB MMRd/NSMP low‐grade endometrioid tumors with absent/focal LVSI, stage IA MMRd/NSMP high‐grade tumors with absent/focal LVSI, and stage IA p53abn tumors without myometrial invasion; high‐intermediate risk comprised stage I MMRd/NSMP tumors with substantial LVSI, stage IB MMRd/NSMP high‐grade tumors, and stage II MMRd/NSMP tumors; high risk comprised stage III–IVA MMRd/NSMP tumors without residual disease and stage I–IVA p53abn or nonendometrioid tumors with myometrial invasion; and advanced/metastatic disease comprised stage III–IVA tumors with residual disease and stage IVB tumors. In accordance with the guidelines, no adjuvant treatment was recommended for low‐risk disease, vaginal brachytherapy for intermediate‐risk disease, external beam radiotherapy with or without chemotherapy for high‐intermediate‐risk disease, chemotherapy with or without external beam radiotherapy for high‐risk disease, and systemic therapy with individualized local treatment for advanced/metastatic disease. The final adjuvant treatment was decided by the institutional multidisciplinary tumor board, and the treatment actually recommended was recorded. The purpose of this stratification was to describe whether the coexistence of adenomyosis was associated with a different distribution of guideline‐defined risk groups and, consequently, with a different allocation of adjuvant therapy.

**FIGURE 2:**
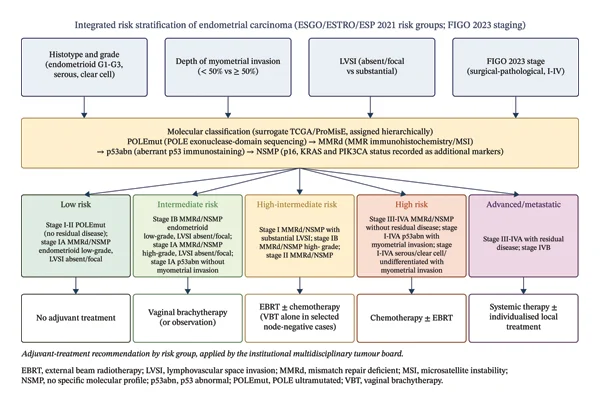
Integrated risk stratification of endometrial carcinoma used in this study. The five prognostic risk groups (low, intermediate, high‐intermediate, high, and advanced/metastatic) were assigned according to the ESGO/ESTRO/ESP 2021 guidelines [25], combining FIGO 2023 stage [27], histotype and grade, depth of myometrial invasion, lymphovascular space invasion, and the surrogate molecular class (POLEmut, MMRd, p53abn, NSMP), assigned hierarchically. The lower row shows the adjuvant treatment recommended for each risk group, as applied by the institutional multidisciplinary tumor board.

### 2.5. Statistical Analysis

Categorical variables are presented as absolute numbers and percentages, and continuous variables as mean and range. Comparisons between patients with and without adenomyosis were performed using Fisher’s exact test for all two‐by‐two contingency tables and the chi‐square test for contingency tables with more than two categories (FIGO stage, histological type and grade). For each dichotomous comparison, the association with adenomyosis is expressed as an unadjusted odds ratio (OR) with a 95% confidence interval (CI), calculated with the Woolf method; when a cell contained zero observations, the Haldane–Anscombe correction (adding 0.5 to each cell) was applied. Variables that were assessed only in a subset of patients (sentinel lymph node status, evaluated only in patients who underwent SLNB; p53 immunostaining, not assessable in seven patients) were analyzed among patients with available data, and the denominators are reported; no imputation of missing data was performed. A two‐sided *p*‐value below 0.05 was considered statistically significant. Because 13 prespecified comparisons were performed, no formal adjustment for multiple testing was applied, but the results were also interpreted against a Bonferroni‐corrected threshold (0.05/13 ≈ 0.004), and the risk of type I error is acknowledged. A multivariable logistic regression analysis to estimate the association of adenomyosis with the main outcomes after adjustment for histotype, grade, stage, and other prognostic factors was considered; however, with only 27 patients in the adenomyosis group and few events in several categories, such a model would have been severely overfitted (fewer than 10 events per candidate covariate), and it was therefore not performed. A post hoc evaluation of the minimum detectable effect size was carried out: with 27 versus 60 patients, a two‐sided alpha of 0.05 and 80% power, the study could detect an OR of approximately 4 or larger (or 0.25 or smaller) for a characteristic present in 25%–50% of patients without adenomyosis, and an OR of approximately 5–6 or larger for characteristics with a baseline prevalence of 7%–12%, such as the individual molecular alterations. Analyses were performed with Python 3 (SciPy 1.17).

## 3. Results

During the study period, 93 patients underwent hysterectomy for EC; 6 were excluded (3 because of an ECOG performance status ≥ 2, 2 because of a final diagnosis of uterine sarcoma, and 1 because IHC and/or molecular profiling was not available), and 87 patients were included in the analysis (Figure 1). Among them, 27 patients (31.0%) had coexisting adenomyosis, while 60 patients (69.0%) did not. The mean age at surgery was 60.5 years (range 38–83) in the adenomyosis group and 61.5 years (range 37–90) in the group without adenomyosis. The histopathological, IHC, and molecular characteristics of the two groups, together with the unadjusted ORs and 95% CIs, are summarized in Table 1.

**TABLE 1:** Comparison of histopathological, immunohistochemical, and molecular characteristics and adjuvant therapy in endometrial cancer with or without coexisting adenomyosis.

Variable	Category	All (*n* = 87)	Adenomyosis (*n* = 27)	No adenomyosis (*n* = 60)	Or (95% CI)^a^	*p* ^b^
Age at surgery, years, mean (range)		61.7 (37–90)	60.5 (38–83)	61.5 (37–90)	—	—

FIGO 2023 stage	I	50 (57.5)	22 (81.5)	28 (46.7)	5.03 (1.68–15.04)	**0.003**
	II	19 (21.8)	3 (11.1)	16 (26.7)		0.025^c^
	III	17 (19.5)	2 (7.4)	15 (25.0)		
	IV	1 (1.1)	0 (0.0)	1 (1.7)		

Histological type and grade	EEC G1	13 (14.9)	8 (29.6)	5 (8.3)	4.63 (1.35–15.90)	**0.019**
	EEC G2	48 (55.2)	16 (59.3)	32 (53.3)	1.27 (0.51–3.19)	0.648
	EEC G3	17 (19.5)	1 (3.7)	16 (26.7)	0.11 (0.01–0.84)	**0.017**
	Serous	7 (8.0)	1 (3.7)	6 (10.0)	0.35 (0.04–3.03)	0.428
	Clear cell	2 (2.3)	1 (3.7)	1 (1.7)	2.27 (0.14–37.7)	0.527
	Overall					0.017^c^

Myometrial invasion	< 50%	39 (44.8)	18 (66.7)	21 (35.0)	3.71 (1.42–9.70)	**0.010**
	≥ 50%	48 (55.2)	9 (33.3)	39 (65.0)		

LVSI	Absent/focal	51 (58.6)	21 (77.8)	30 (50.0)	3.50 (1.24–9.89)	**0.019**
	Substantial	36 (41.4)	6 (22.2)	30 (50.0)		

Sentinel lymph node status^d^	Negative	40/43 (93.0)	10/12 (83.3)	30/31 (96.8)	6.00 (0.49–73.5)^e^	0.184
	Positive	3/43 (7.0)	2/12 (16.7)	1/31 (3.2)		

Pelvic/para‐aortic lymphadenectomy	Yes	39 (44.8)	11 (40.7)	28 (46.7)	0.79 (0.31–1.97)	0.648
	No	48 (55.2)	16 (59.3)	32 (53.3)		

p53 immunostaining^f^	Wild‐type	73/80 (91.3)	26/26 (100)	47/54 (87.0)	0.12 (0.01–2.18)^g^	0.090
	Abnormal	7/80 (8.8)	0/26 (0.0)	7/54 (13.0)		

p16 immunostaining	Positive	75 (86.2)	21 (77.8)	54 (90.0)	0.39 (0.11–1.34)^h^	0.178
	Negative	12 (13.8)	6 (22.2)	6 (10.0)		

Molecular markers	POLE‐mutated	7 (8.0)	3 (11.1)	4 (6.7)	1.75 (0.36–8.42)	0.672
	MMR‐deficient	20 (23.0)	5 (18.5)	15 (25.0)	0.68 (0.22–2.12)	0.591
	KRAS‐mutated	17 (19.5)	4 (14.8)	13 (21.7)	0.63 (0.18–2.14)	0.567
	PIK3CA‐altered	41 (47.1)	13 (48.1)	28 (46.7)	1.06 (0.43–2.64)	1.000

Adjuvant therapy^i^	Yes	43 (49.4)	8 (29.6)	35 (58.3)	0.30 (0.11–0.80)	**0.020**
	No	44 (50.6)	19 (70.4)	25 (41.7)		

*Note:* Data are *n* (%) unless otherwise stated; percentages are calculated on the number of patients with available data. Statistically significant *p*‐values (*p* < 0.05) are shown in bold. LVSI, lymphovascular space invasion; MMR, mismatch repair.

Abbreviations: CI, confidence interval; EEC, endometrioid endometrial carcinoma; FIGO, International Federation of Gynecology and Obstetrics; OR, odds ratio.

^a^Unadjusted odds ratio (adenomyosis vs. no adenomyosis) for the category listed first in each variable, or for the category indicated, calculated with the Woolf method.

^b^Fisher’s exact test unless otherwise indicated.

^c^Chi‐square test for the overall distribution across categories.

^d^Assessed only in the 43 patients who underwent sentinel lymph node biopsy (12 with and 31 without adenomyosis); OR for a positive sentinel node.

^e^Wide confidence interval owing to very few events.

^f^Not assessable in 7 patients (1 with and 6 without adenomyosis).

^g^OR for abnormal p53, Haldane–Anscombe correction applied because of a zero cell.

^h^OR for p16 negativity: 2.57 (0.75–8.88).

^i^Vaginal brachytherapy, external beam pelvic radiotherapy, chemotherapy, or combined modality.

Regarding FIGO staging, EC with adenomyosis was more frequently diagnosed at stage I (22/27, 81.5%) than EC without adenomyosis (28/60, 46.7%; OR 5.03, 95% CI 1.68–15.04; *p* = 0.003). Conversely, stage III disease was present in 7.4% of cases with adenomyosis and 25.0% of cases without, while the only case of stage IV disease occurred in the group without adenomyosis (1.7%); the overall distribution of stages differed between groups (*p* = 0.025). Histopathological analysis showed a different distribution of histotype and grade (*p* = 0.017): grade 1 endometrioid EC (EEC G1) was more frequent in the adenomyosis group (29.6% vs. 8.3%; OR 4.63, 95% CI 1.35–15.90; *p* = 0.019), whereas grade 3 endometrioid tumors (EEC G3) were less frequent (3.7% vs. 26.7%; OR 0.11, 95% CI 0.01–0.84; *p* = 0.017). Nonendometrioid histotypes (serous and clear cell carcinoma) were observed in 2/27 (7.4%) and 7/60 (11.7%) patients, respectively (*p* = 0.715). Myometrial invasion of less than 50% of the myometrial thickness was more frequent in the adenomyosis group (66.7% vs. 35.0%; OR 3.71, 95% CI 1.42–9.70; *p* = 0.010), and LVSI was absent or focal in 77.8% of EC cases with adenomyosis compared with 50.0% of those without (OR 3.50, 95% CI 1.24–9.89; *p* = 0.019).

SLNB was performed in 43 patients (12 with and 31 without adenomyosis); the proportion of positive sentinel nodes did not differ between groups (16.7% vs. 3.2%; *p* = 0.184), although the estimate was very imprecise (OR 6.00, 95% CI 0.49–73.5). Pelvic/para‐aortic lymphadenectomy was performed in 40.7% and 46.7% of patients, respectively (*p* = 0.648). p53 immunostaining was assessable in 80 patients: an abnormal pattern was found in none of the 26 assessable patients with adenomyosis and in 7/54 (13.0%) of those without (OR 0.12, 95% CI 0.01–2.18; *p* = 0.090). p16 negativity (22.2% vs. 10.0%; *p* = 0.178) and the prevalence of POLE mutations (11.1% vs. 6.7%; OR 1.75, 95% CI 0.36–8.42; *p* = 0.672), MMR deficiency (18.5% vs. 25.0%; OR 0.68, 95% CI 0.22–2.12; *p* = 0.591), KRAS mutations (14.8% vs. 21.7%; OR 0.63, 95% CI 0.18–2.14; *p* = 0.567), and PIK3CA alterations (48.1% vs. 46.7%; OR 1.06, 95% CI 0.43–2.64; *p* = 1.000) did not differ significantly between groups; for all these markers, the CIs were wide and compatible with both substantial increases and decreases in prevalence.

Adjuvant therapy was recommended for 8/27 (29.6%) patients with adenomyosis and 35/60 (58.3%) patients without (OR 0.30, 95% CI 0.11–0.80; *p* = 0.020). When the results were interpreted against the Bonferroni‐corrected threshold (*p* < 0.004), only the association with FIGO stage I remained statistically significant.

## 4. Discussion

In this single‐center retrospective cohort of 87 women who underwent hysterectomy for EC with complete IHC and molecular characterization, coexisting adenomyosis was found in 31% of specimens and was associated with a more favorable pathological profile: EC with adenomyosis was more frequently diagnosed at FIGO stage I, was more often of low grade, showed less deep myometrial invasion and less substantial LVSI, all features that are established determinants of prognosis and of adjuvant treatment [25, 28]. No significant differences were observed in nodal status, p53 and p16 expression, or in the molecular markers assessed (POLE, MMR, KRAS, and PIK3CA). Consistently with these pathological differences, patients with adenomyosis were less frequently allocated to adjuvant therapy. These findings describe associations observed in this cohort; as discussed below, the study design does not allow us to determine whether adenomyosis is independently related to tumor features or whether it merely co‐occurs with them.

The prevalence of adenomyosis observed in the present series is in line with the literature. A meta‐analysis of 5573 EC patients found a pooled prevalence of adenomyosis of 22.6% in hysterectomy specimens [6]. Given its high prevalence, several studies have investigated its potential impact on prognosis. A meta‐analysis by An et al., which included retrospective observational studies, reported a significantly higher overall survival in EC patients with adenomyosis [14], and a further meta‐analysis of 2505 EC patients, of whom 553 had adenomyosis, found approximately half the risk of death and recurrence in patients with adenomyosis compared with those without [17]. Several biological mechanisms have been proposed to explain these observations, including a cytokine milieu that is less favorable to tumor growth and invasion, and structural changes of the endometrial–myometrial interface that could act as a mechanical barrier to myometrial infiltration [3, 14]. It should be emphasized, however, that these are hypotheses that our data cannot test. Indeed, the same meta‐analysis noted that the impact of adenomyosis on mortality could not be assessed in multivariable analyses and that the better prognosis observed at univariate analysis may not be independent, but rather explained by the favorable prognostic factors that are more frequent in EC with adenomyosis [17]. Chou et al. explicitly argued that the good outcome of EC patients with adenomyosis is a consequence of their associated favorable clinicopathological parameters rather than of adenomyosis per se [16]. The present findings are entirely compatible with this interpretation: the differences we observed concern precisely those clinicopathological features—stage, grade, myometrial invasion and LVSI—which are strongly interrelated, and our univariate analysis cannot disentangle them.

The lower frequency of adjuvant therapy among patients with adenomyosis deserves a specific comment. In our institution, as in most centers, adjuvant treatment is allocated by the multidisciplinary tumor board according to guideline‐defined risk groups that are determined by stage, histotype and grade, depth of myometrial invasion, LVSI, and molecular class [25]. Adenomyosis is not a criterion in this algorithm. The reduced allocation to adjuvant therapy in the adenomyosis group is therefore most plausibly a direct consequence of the more favorable pathological characteristics observed in these patients, which placed a larger proportion of them in the low‐risk group, and should not be interpreted as evidence that adenomyosis itself influences treatment decisions or tumor biology. In other words, the adjuvant‐therapy finding is a downstream reflection of the pathological findings rather than an independent result.

The absence of significant differences in the IHC and molecular markers also requires careful interpretation. The prevalence of POLE mutations, MMR deficiency, KRAS, and PIK3CA alterations was similar in the two groups, which is consistent with the multicentric exploratory analysis by Raimondo et al., who found no distinctive molecular signature in EC with coexistent adenomyosis [9]. A true biological similarity between EC with and without adenomyosis is therefore a plausible interpretation. However, these null results cannot be taken as evidence of equivalence. The individual molecular alterations were present in only 7 to 20 patients overall, the CIs of the corresponding ORs were very wide (e.g., 0.36–8.42 for POLE and 0.22–2.12 for MMR deficiency), and, as shown by our post hoc evaluation, the study could only detect differences corresponding to an OR of approximately 4–6 for markers with a baseline prevalence below 25%. Smaller but clinically meaningful differences cannot be excluded (type II error). Similarly, the complete absence of p53‐abnormal tumors in the adenomyosis group (0/26 vs. 7/54) is a potentially interesting observation, but it did not reach statistical significance and is based on very few events.

One of the strengths of this study is the comparative approach, which allowed a direct assessment of differences between EC cases with and without adenomyosis within a single institution, with uniform surgical management, pathological work‐up, and adjuvant treatment policy. Unlike several previous studies, which focused primarily on histopathological parameters, this study integrated histological, IHC, and molecular features within the framework of contemporary FIGO staging and guideline‐based risk stratification, and specifically examined adjuvant therapy allocation, a clinically relevant outcome that has been insufficiently addressed. Another notable strength is the inclusion of both endometrioid and nonendometrioid histotypes, and the use of direct molecular analysis for MMR status and POLE sequencing rather than IHC surrogates alone.

The study has several important limitations. First, the retrospective, single‐center design precludes causal inference, and data collection relied on previously recorded clinical and pathological information. Second, and most importantly, the statistical analysis was limited to univariate comparisons. With only 27 patients in the adenomyosis group and few events in several categories, a multivariable logistic regression adjusting for grade, histotype, stage, and other prognostic factors could not be reliably fitted, and we deliberately chose not to report estimates from an overfitted model. Consequently, we cannot establish whether adenomyosis is independently associated with favorable pathological characteristics or whether the observed associations reflect differences in baseline disease characteristics; our results should be regarded as descriptive and hypothesis‐generating. Third, the sample size limited the statistical power, particularly for molecular subgroup analyses, as discussed above. Fourth, 13 comparisons were performed without adjustment for multiplicity; when a Bonferroni‐corrected threshold was applied, only the association with FIGO stage I remained significant, and the remaining associations should be interpreted with caution. Fifth, the study did not evaluate recurrence, disease‐free, progression‐free, or overall survival; because the study period was relatively short (January 2023 to September 2024) and follow‐up is still limited, no conclusion regarding prognosis can be drawn from our data, and our findings are restricted to pathological characteristics and treatment allocation. Sixth, the inclusion of only patients with complete IHC and molecular profiling, and the exclusion of patients with incomplete staging, may have introduced selection bias if excluded patients differed systematically from those analyzed; however, only 6 of 93 patients (6.5%) were excluded, and the study flow is reported in Figure 1 to allow readers to appraise this issue. Seventh, the diagnosis of adenomyosis was made during routine pathological reporting and was not subject to a study‐specific consensus review, and the extent of adenomyosis and its relationship with the tumor (adjacent, involved, or arising within adenomyosis) were not systematically recorded. Finally, p53 immunostaining was not assessable in seven patients, and sentinel lymph node status was, by design, available only in the subset of patients who underwent SLNB.

In summary, our findings are in agreement with existing evidence indicating that EC cases with coexisting adenomyosis tend to display lower‐risk pathological features [14–18]. Whether adenomyosis has any direct biological effect on tumor behavior, or whether its presence is simply correlated with favorable tumor characteristics—for example, through shared hormonal or patient‐related factors—cannot be determined from this or from other retrospective series. Future prospective, multicenter studies with larger cohorts, adequate statistical power, multivariable analysis, systematic characterization of the adenomyosis–tumor relationship, and survival outcomes are needed before the coexistence of adenomyosis can be considered in risk stratification or in clinical decision‐making.

## 5. Conclusion

In this single‐center retrospective cohort, EC with coexisting adenomyosis was associated, at univariate analysis, with earlier FIGO stage, lower tumor grade, less deep myometrial invasion, and less substantial LVSI, and consequently with less frequent allocation to adjuvant therapy, whereas no differences were detected in nodal status or in IHC and molecular markers. These observations are consistent with previous reports of a more favorable pathological profile in EC with adenomyosis. However, given the small sample size, the absence of multivariable adjustment and of survival data, and the retrospective design, they do not demonstrate an independent or protective effect of adenomyosis and should not influence clinical decision‐making. Validation in larger, prospective, multicenter studies is required.

## Author Contributions

Conceptualization, Marco D’Asta; methodology, Marco D’Asta; software, Ferdinando Antonio Gulino and Giosuè Giordano Incognito; validation, Carla Ettore; formal analysis, Ferdinando Antonio Gulino and Giosuè Giordano Incognito; investigation, Marco D’Asta, Caterina Monaco, Carla Ettore, and Giuseppe Ettore; resources, Giuseppe Ettore; data curation, Marco D’Asta, Caterina Monaco, Carla Ettore, and Giuseppe Ettore; writing–original draft, Marco D’Asta, Caterina Monaco, and Ferdinando Antonio Gulino; writing–review and editing, Carla Ettore, Giosuè Giordano Incognito, and Giuseppe Ettore; visualization, Carla Ettore and Giosuè Giordano Incognito; supervision, Giuseppe Ettore; project administration, Giuseppe Ettore.

## Funding

This research received no specific grant from any funding agency in the public, commercial, or not‐for‐profit sectors.

## Disclosure

The manuscript guarantor, Ferdinando Antonio Gulino, affirms that this manuscript is an honest, accurate, and transparent account of the study being reported; that no important aspects of the study have been omitted; and that any discrepancies from the study as planned have been explained. All authors have read and approved the final version of the manuscript. Ferdinando Antonio Gulino (corresponding author and manuscript guarantor) had full access to all of the data in this study and takes complete responsibility for the integrity of the data and the accuracy of the data analysis. No financial relationships relevant to this work exist that could have influenced the study design; the collection, analysis, and interpretation of data; the writing of the report; or the decision to submit the report for publication.

## Ethics Statement

The study was conducted in accordance with the Declaration of Helsinki and is reported following the Strengthening the Reporting of Observational Studies in Epidemiology (STROBE) Statement. Owing to the retrospective design and the use of anonymized data obtained during routine clinical care, the requirement for study‐specific informed consent was waived; all patients had provided written informed consent for surgery and for the use of their anonymized clinical data for research purposes at the time of hospital admission.

## Conflicts of Interest

The authors declare no conflicts of interest.

## Data Availability

The aggregated data supporting the findings of this study are available within the article and its figures. The individual patient‐level data are not publicly available because they contain information that could compromise the privacy of research participants, but are available from the corresponding author upon reasonable request, subject to approval by the institutional Ethics Committee.
